# The Identification of Plasma Exosomal miR-423-3p as a Potential Predictive Biomarker for Prostate Cancer Castration-Resistance Development by Plasma Exosomal miRNA Sequencing

**DOI:** 10.3389/fcell.2020.602493

**Published:** 2021-01-07

**Authors:** Tianyu Guo, Yang Wang, Jing Jia, Xueying Mao, Elzbieta Stankiewicz, Glenda Scandura, Edwina Burke, Lei Xu, Jacek Marzec, Caitlin R. Davies, Jiaying Jasmin Lu, Prabhakar Rajan, Alistair Grey, Karen Tipples, John Hines, Sakunthala Kudahetti, Tim Oliver, Thomas Powles, Constantine Alifrangis, Manish Kohli, Greg Shaw, Wen Wang, Ninghan Feng, Jonathan Shamash, Daniel Berney, Liang Wang, Yong-Jie Lu

**Affiliations:** ^1^Centre for Cancer Biomarker and Biotherapeutics, Barts Cancer Institute, Queen Mary University of London, London, United Kingdom; ^2^Department of Cell Biology, Zhejiang University School of Medicine, The Second Affiliated Hospital, Hangzhou, China; ^3^Department of Urology, Affiliated Wuxi No. 2 Hospital of Nanjing Medical University, Wuxi, China; ^4^Department of Tumor Biology, H. Lee Moffitt Cancer Center, Tampa, FL, United States; ^5^Department of Urology, Zhongshan Hospital, Fudan University, Shanghai, China; ^6^Centre for Cancer Research, University of Melbourne, Melbourne, VIC, Australia; ^7^Department of Urology, Barts Health NHS, London, United Kingdom; ^8^Division of Surgery and Interventional Sciences, University College London, London, United Kingdom; ^9^Department of Uro-oncology, University College London NHS Foundation Trust, London, United Kingdom; ^10^Department of Medicine, University of Utah, Huntsman Cancer Institute, Salt Lake City, UT, United States; ^11^Department of Oncology, Mayo Clinic, Rochester, MN, United States; ^12^Division of Bioengineering, School of Engineering and Materials Science, Queen Mary University of London, London, United Kingdom; ^13^Department of Medical Oncology, Barts Health NHS, London, United Kingdom

**Keywords:** prostate cancer, castration-resistance development, biomarker, plasma exosome miRNA, miR-423-3p

## Abstract

Castration-resistant prostate cancer (CRPC) is the major cause of death from prostate cancer. Biomarkers to improve early detection and prediction of CRPC especially using non-invasive liquid biopsies could improve outcomes. Therefore, we investigated the plasma exosomal miRNAs associated with CRPC and their potential for development into non-invasive early detection biomarkers for resistance to treatment. RNA-sequencing, which generated approximately five million reads per patient, was performed to identify differentially expressed plasma exosomal miRNAs in 24 treatment-naive prostate cancer and 24 CRPC patients. RT-qPCR was used to confirm the differential expressions of six exosomal miRNAs, miR-423-3p, miR-320a, miR-99a-5p, miR-320d, miR-320b, and miR-150-5p (*p* = 7.3 × 10^−8^, 0.0020, 0.018, 0.0028, 0.0013, and 0.0058, respectively) firstly in a validation cohort of 108 treatment-naive prostate cancer and 42 CRPC patients. The most significant differentially expressed miRNA, miR-423-3p, was shown to be associated with CRPC with area under the ROC curve (AUC) = 0.784. Combining miR-423-3p with prostate-specific antigen (PSA) enhanced the prediction of CRPC (AUC = 0.908). A separate research center validation with 30 treatment-naive and 30 CRPC patients also confirmed the differential expression of miR-423-3p (*p* = 0.016). Finally, plasma exosomal miR-423-3p expression in CRPC patients was compared to 36 non-CRPC patients under androgen depletion therapy, which showed significantly higher expression in CRPC than treated non-CRPC patients (*p* < 0.0001) with AUC = 0.879 to predict CRPC with no difference between treatment-naive and treated non-CRPC patients. Therefore, our findings demonstrate that a number of plasma exosomal miRNAs are associated with CRPC and miR-423-3p may serve as a biomarker for early detection/prediction of castration-resistance.

## Introduction

Prostate cancer (PCa) is the most frequently diagnosed male cancer and the second-leading cause of oncological mortality in the USA, with estimated 174,650 new cases and 31,620 deaths in 2019 (Siegel et al., 2019). Androgen deprivation therapy (ADT) has been the standard of care for initial management of locally advanced and metastatic PCa. However, patients inevitably progress to castration-resistant PCa (CRPC) within 1–3 years from the start of primary ADT, despite the initial benefits (Chandrasekar et al., 2015). The prognosis of CRPC patients is historically poor, with median overall survival after ADT failure being 2–3 years (West et al., 2014; Ryan et al., 2015). Prognostic biomarkers for patients with CRPC have been investigated in many studies (Armstrong et al., 2012; Olmos et al., 2012; Ross et al., 2012; Huang et al., 2015; Pantel et al., 2019) and circulating tumor cell analysis has been approved by the FDA as a prognostic biomarker for patients with metastatic CRPC (Pantel et al., 2019). In contrast, biomarkers to predict or monitor CRPC development are rarely investigated (Varenhorst et al., 2016), despite the fact that prediction and early detection of CRPC and earlier modifications to treatment may be more effective in controlling the disease than changing therapy after clinically apparent CRPC has developed.

Currently, CRPC is usually diagnosed based on biochemical and radiographic progression (Cornford et al., 2017). Biochemical progression relies on measuring the serum prostatic-specific antigen (PSA) level. However, the serum PSA level does not always correlate with the clinical status of CRPC (Mizokami et al., 2017). Radiographic progression reflects well-developed CRPC and necessitates frequent bone and CT scans. These issues may lead to delays in treatment changes, missing the opportunity to eradicate small subclones of CRPC cells when it is easier to cure. Thus, it is important to further investigate the molecular mechanisms of CRPC development and identify additional liquid biopsy biomarkers, which can be more easily utilized to efficiently detect/predict early CRPC to promptly change treatment into one of the effective therapies developed in recent years for CRPC patients (Afshar et al., 2015).

Exosomes are small cell secreted vesicles (30–150 nm), which contain numerous molecular constituents, including lipids, proteins, RNA and DNA, and mediate cell-cell communication by transferring these exosomal components between cells (Rana et al., 2013; Matei et al., 2017; Chen et al., 2018). MicroRNAs (miRNAs) are enriched in exosomes (Valadi et al., 2007) and can be transferred between cells via exosomes to regulate various biological processes associated with cancer development and progression (Rana et al., 2013; Sánchez et al., 2016). MiRNAs in exosomes are protected from degradation in the circulation (Ge et al., 2014) and studies have demonstrated the potential of exploiting exosomal miRNAs as non-invasive and dynamic biomarkers in cancer diagnosis and prognosis (Hu et al., 2012). Plasma exosomal miRNAs have been previously reported as valuable prognostic biomarkers in patients who have already developed CRPC (Huang et al., 2013, 2015; Yuan et al., 2016). However, no studies on the association of plasma exosomal miRNA with CRPC development have been reported.

Identification of plasma exosomal miRNAs associated with CRPC would not only improve our understanding of CRPC development mechanisms, but more importantly help the development of biomarkers to predict/detect the early occurrence of CRPC, enabling prompt alteration of therapeutic regimens before CRPC is fully developed. We therefore investigated plasma exosomal miRNAs associated with CRPC and evaluated their potential as predictive biomarkers for CRPC occurrence.

## Materials and Methods

### Patients

**Figure 1:**
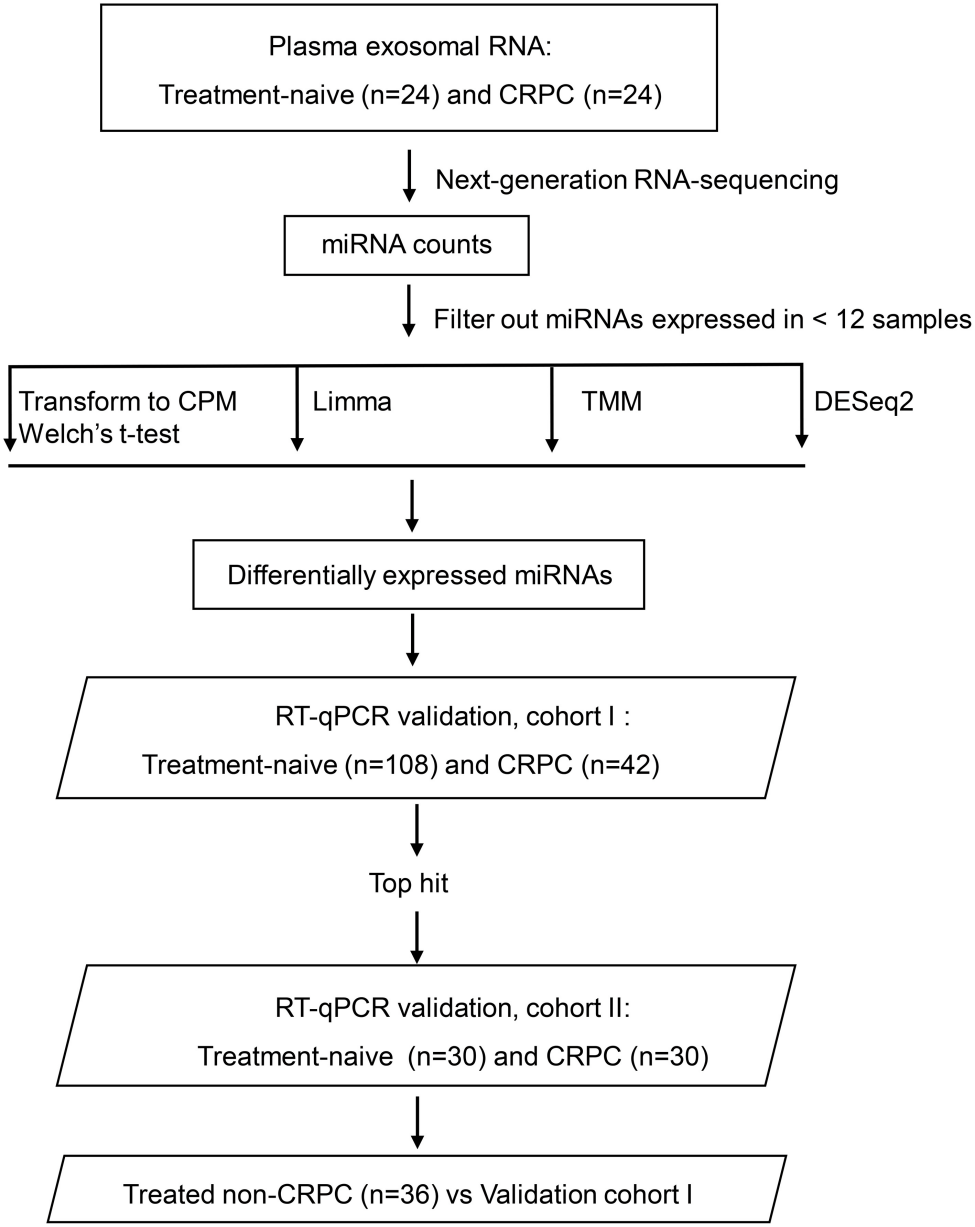
Overview of the plasma exosomal miRNA analysis workflow. CRPC, castration-resistant prostate cancer; CPM, counts per million transformation; Limma, linear models for microarray and RNA-sequencing data; TMM, the trimmed mean of M-value normalization.

Blood samples from 24 treatment-naive PCa patients and 24 CRPC patients for RNA next-generation sequencing (RNA-seq) and 108 treatment-naive PCa and 42 CRPC patients (including the 24 CRPC patients used for RNA-seq) in the validation cohort I were collected with patients' informed consent from St Bartholomew's Hospital, BartsHealth NHS, London, UK, between 2015 and 2018. Samples from an additional cohort of treated non-CRPC patients included 36 PCa patients who had started initial hormone-therapy <3 months before blood collection was obtained also from St Bartholomew's Hospital. Blood samples from 30 treatment-naive PCa patients and 30 CRPC patients in the independent validation cohort II were collected from Mayo Clinic, Rochester, US between 2009 and 2012 with patients' informed consent. All CRPC patients had been treated with ADT and had evidence of disease progression with rising PSA and/or imaging-based progression. Patients' clinical data is summarized in Table 1. Use of patient blood samples and clinical data in this study was approved by London City & East Research Ethics Committee (09/H0704/4+5) and Institutional Review Board of Medical College of Wisconsin (PRO00017780). RNA-seq and validation workflow is presented in Figure 1.

**Table 1:** Clinical characteristics of patients in the RNA-sequencing and RT-qPCR validation cohorts.

	**RNA-sequencing cohort**		**Validation cohort I**		**Treated non-CRPC**	**Validation cohort II**	
	**Treatment-naive**	**CRPC**	**Treatment-naive**	**CRPC^a^**		**Treatment-naive**	**CRPC**
Case number	24	24	108	42	36	30	30
Age, yr, median (IQR)	65.15 (56.13-70.73)	75.55 (68.75-81.89)	65.90 (57.89–72.10)	72.65 (68.38–80.88)	65.90 (61.10–73.55)	67.30 (63.38–80.55)	71.70 (65.85–76.58)
**Gleason score at diagnosis**							
≤6	8	1	36	1	1	2	4
7	16	2	58	7	11	14	9
8	0	5	6	8	5	3	4
≥9	0	9	8	16	14	8	10
unknown	0	7	0	10	5	3	3
Median PSA at sample collection, ng/ml (IQR)	9.9 (6.9–12.75)	51 (17.75–241.8)	8.9 (5.75–13)	57 (21.5–192.3)	15.5 (6.255–112.7)	7 (1.75–28.55)^b^	47.7 (9.35–119)

*Yr, year; IQR, interquartile range; CRPC, castration-resistant prostate cancer; PSA, prostate-specific antigen*.

a including 24 patients used for RNA-sequencing;

b *one patient's data is missing*.

### Exosome Isolation

Plasma was isolated within 2 h of blood draw by centrifugation of whole blood at 1,200 g for 10 min, followed by another centrifugation of the supernatant at 2,000 g for 10 min. The supernatant was taken as plasma and stored at −80°C for future use. Two hundred microliter of plasma from each case was used. Prior to exosome precipitation, plasma samples were treated with RNase A to remove free circulating RNAs and then RNase A was inactivated with RNase inhibitor. Exosomes were isolated using the Total Exosome Isolation Kit (from plasma) (Invitrogen™) following manufacturer's instructions. For the validation cohort at Mayo Clinic, US, exosomes were isolated using ExoQuick Plasma prep and Exosome precipitation kit (System Biosciences) without RNase pre-treatment.

### RNA Extraction

The exosome pellet was re-suspended in Buffer RLT (Qiagen) with 0.25 μg/μl Proteinase K and incubated at 50°C for 30 min. Exosomal RNA was then extracted using miRNeasy Micro Kit (Qiagen) and QIAzol Lysis Reagent (Qiagen) according to the manufacturer's protocol. PC3 cell miRNAs were extracted using AllPrep® DNA/RNA/Protein Mini kit (Qiagen) and miReasy micro kit (Qiagen) according to the recommended protocol.

### RNA-Seq and Data Analysis

Indexed libraries were prepared from the exosomal RNA as instructed by the NEBNext Multiplex Small RNA Library Prep Set for Illumina (NEB) without size selection. RNA-seq was performed on Illumina NextSeq 500 platform. The data was trimmed with trimgalore (end minimum quality level 30 and minimum read length 15) and aligned to human reference genome build hg19 with Bowtie 0.12.8 (seed mismatch limit 1 and seed length 10) implemented in BaseSpace (Illumina, CA). BAM files were uploaded into Partek Genomic Suite (Partek Inc) and annotated against mirBase 20 mature miRNA. Samples were sequenced twice and read counts from two runs were combined for data analysis. The raw data was deposited to Gene Expression Omnibus (accession number: GSE136321). MiRNAs expressed in <25% of samples were removed from differential expression analysis. The miRNA read counts were analyzed using four pipelines to identify differentially expressed miRNAs (Figure 1): (1) Counts per million (CPM), (2) The trimmed mean of M-value normalization (TMM) (Robinson and Oshlack, 2010), (3) Limma (Ritchie et al., 2015), and (4) DESeq2 (Love et al., 2014). For CPM analysis, filtered read counts were transformed to CPM using the cpm() function implemented in edgeR package (version 2.4.0) and then Welch's *t*-test was performed in SPSS 24. TMM was performed using functions implemented in the edgeR. Filtered read counts were input as a DGEList in R (v4.0.2). TMM normalization was performed using calcNormFactors() function implemented in edgeR() package. EstimateCommonDisp() was performed to estimate the common dispersion parameter and the ExactTest() functions in edgeR were used to detect differentially expressed miRNAs. The results were then written out as a csv file. Pipeline combining functions in Limma and edgeR were performed using functions implemented in both software packages in R. Filtered read counts were input as a DGEList and normalized with calcNormFactors() function. The normalized data was voom transformed with voom() function. Then a linear model was fitted using lmfit() and the empirical Bayes statistics was applied with eBayes() function to smooth the standard errors. The results were then written out as a csv file. DESeq2 differential expression analysis was performed using functions in the DESeq2 R package (v4.0.2). A DESeq data set was generated with filtered read counts using DESeqDataSetFromMatrix() function implemented in DESeq2() package. Normalization and differentially expressed gene analysis was done using DESeq() function. The results were then written out as a csv file.

### Reverse Transcription Quantitative Real-Time Polymerase Chain Reaction (RT-qPCR)

The reverse transcription was performed using miScript II RT kit (Qiagen). RT-qPCR was performed with miScript primer assays (Qiagen) (MS00004179—Hs_miR-423_1 miScript Primer, MS00008932—Hs_miR-193a-5p_1 miScript Primer, MS00003738—Hs_miR-200a_1 miScript Primer, MS00032158—Hs_miR-99a_2 miScript Primer, MS00014707—Hs_miR-320a_1 miScript Primer, MS00031710—Hs_miR-320d_2 miScript Primer, MS00031703—Hs_miR-320b_2 miScript Primer, MS00006552—Hs_miR-24_1 miScript Primer, MS00010752—Hs_miR-9_1 miScript Primer, MS00007350—Hs_miR-30a-5p_1 miScript Primer, MS00003129—Hs_let-7c_1 miScript Primer, MS00008372—Hs_miR-101_3 miScript Primer, MS00003556—Hs_miR-148a_1 miScript Primer, MS00003577—Hs_miR-150_1 miScript Primer, MS00031829—Hs_miR-375_2 miScript Primer, MS00004242—Hs_miR-451_1 miScript Primer) and miScript SYBR® Green PCR Kit (Qiagen) on Quantstudio^TM^ real-time PCR system (ThermoFisher Scientific). Let-7c-5p and miR-30a-5p were selected as endogenous reference genes based on their small variation across all samples in our sequencing data and previous publication (Huang et al., 2015). ΔCt = (Ct^miRNA^ – average Ct^referencegenes^) and where PC3 cell line miRNA was used to normalize multiple RT-qPCR plates, ΔCt was calculated as sample (Ct^miRNA^ – average Ct^referencegenes^) – PC3 (Ct^miRNA^ – average Ct^referencegenes^). The relative expression level of miRNAs in exosomes was calculated as 2^−ΔCt^. All reactions were run in triplicate.

### Pathway Over-Representation Analysis

Targets of miRNAs were retrieved from TarBase v7.0 and pathway overrepresentation analysis was performed using KEGG pathways via online tool DIANA-miRPath v3.0 (http://snf-515788.vm.okeanos.grnet.gr/) (Vlachos et al., 2015). The CRPC associated miRNAs identified in this study were input and analyzed using TarBase to retrieve target genes of the miRNAs. Pathways union mode was used to identify all the pathways significantly targeted by the selected miRNAs. False Discovery Rate correction was applied. *P*-value threshold was set at 0.05 and microT threshold was defaulted at 0.8. Fisher's exact test was selected as the enrichment analysis method. To generate heatmap for each miRNA, significance clusters/heatmap option was selected.

### Statistical Analysis

Analysis of the RNA-seq data was described in previous section. Unpaired Mann-Whitney U tests were performed on RT-qPCR relative expression levels to identify differences in exosomal miRNA expression levels between treatment-naive PCa, treated non-CRPC and CRPC and were performed in GraphPad Prism 7. Receiver operating characteristic (ROC) curves were generated and the area under the curve (AUC) was used to evaluate the prediction values of parameters for CRPC. ROC curve and AUC was generated in GraphPad Prism 7. Binomial logistic regression was performed with miR-423-3p relative expression level and PSA level as predictors for CRPC (yes, no) using the binomial logistic regression function in SPSS 24. A combination model (CM) was computed as the linear predictor of the fitted bivariate logistic model with PSA and 423-3p relative expression level as only predictors as a*PSA+b*miR-423-3p, where the values of “a” and “b” are the covariance factors of PSA and miR-423-3p relative expression level, respectively. Univariate logistic regression analyses were performed separately for miR-423-3p relative expression level and PSA level to evaluate and compare their association with CRPC. The miR-423-3p relative expression and PSA level were treated as continuous variables, and CRPC status was considered as a categorical variable. To examine the independent association of miR-423-3p expression with CRPC status and adjust it for PSA level a multivariate logistic regression analysis was performed. All statistical tests were two sided. We did not apply multiple event testing correction to exosomal RNA-seq data analysis, since there are only a small number of miRNAs for the analysis and the differential miRNA expression was identified by different analysis tools to select candidates (more than selected based on adjusted *p*-value) for experimental validation. Bonferroni correction test was performed to modify *p*-values for RT-qPCR result multiple tests through dividing the critical *p*-value by the number of comparisons being made.

## Results

### The Plasma Exosomal miRNA Expression Profiles in Patients With Treatment-Naive PCa and CRPC

To identify candidate plasma exosomal miRNAs associated with CRPC development, RNA-seq was performed in a screening cohort of 24 treatment-naive PCa patients and 24 CRPC patients. Combing two runs of data, the RNA-seq produced an average of five million reads per sample. This data enabled us to detect 612 plasma exosomal miRNAs in total, with 483 detected in treatment-naive PCa and 499 in CRPC patients (Supplementary Table 1). There were 37 miRNAs which were consistently detected in all individual samples, while 45 miRNAs detected in all CRPC samples and 45 miRNAs detected in all treatment-naive PCa samples (Supplementary Table 2). On average, miR-451a was the most abundant miRNA in both treatment-naive PCa and CRPC. The top ten most abundant miRNAs detected in these two groups are listed in Supplementary Table 3.

### Identification of Differential Expression of miRNAs by RNA-Seq Comparing Patients With Treatment-Naive PCa and CRPC

To identify differentially expressed plasma exosomal miRNAs between the treatment-naive PCa and CRPC patients, we employed four methods (CPM, TMM, Limma, and DESeq2) to analyze the RNA-seq data. MiRNAs expressed in <25% of samples were filtered out from the analysis. From the remaining 185 miRNAs, we identified 13, 13, 11, and 14 differentially expressed miRNAs by Limma, TMM, CPM, and DESeq2 methods, respectively, at *p* < 0.01 (Table 2 and Supplementary Table 4). Among them, five miRNAs (miR-423-3p, miR-99a-5p, miR-320a, miR-200a-3p, and miR-193a-5p) were identified by all four methods and they were all present at higher levels in CRPC than treatment naive PCa patient samples. Six miRNAs (miR-375, miR-451a, miR-320b, miR-148a-3p, miR-150-5p, miR-320d) were detected by three of the methods and three miRNAs (miR-101-3p, miR-9-5p, miR-24-3p) by two of the methods. Of note, all of the miRNAs with *p* < 0.01 from DESeq2 were also detected by one of the other methods. Except for DESeq2, the other three methods presented some unique miRNAs which were not detected by others.

**Table 2:** Differentially expressed plasma exosomal miRNAs at *p* < 0.01 between treatment-naive prostate cancer and CRPC by RNA-sequencing analyses.

**Limma**		**TMM**		**CPM**		**DESeq2**	
**MiRNA**	***p*-value**	**MiRNA**	***p*-value**	**MiRNA**	***p*-value**	**MiRNA**	***p*-value**
miR-375	3.73 × 10^−04^	miR-375	5.71 × 10^−11^	**miR-423-3p**	4.20 × 10^−04^	miR-375	7.79 × 10^−07^
**miR-193a-5p**	3.93 × 10^−04^	**miR-193a-5p**	1.32 × 10^−05^	miR-21-5p	1.06 × 10^−03^	**miR-193a-5p**	2.36 × 10^−05^
miR-101-3p	9.07 × 10^−04^	miR-9-5p	1.56 × 10^−05^	**miR-320a**	1.10 × 10^−03^	miR-320b	1.53 × 10^−04^
miR-451a	1.70 × 10^−03^	miR-184	9.39 × 10^−05^	**miR-193a-5p**	2.07 × 10^−03^	**miR-200a-3p**	2.73 × 10^−04^
miR-320b	1.73 × 10^−03^	**miR-200a-3p**	4.00 × 10^−04^	miR-451a	2.61 × 10^−03^	**miR-320a**	2.83 × 10^−04^
**miR-320a**	3.07 × 10^−03^	miR-320b	4.90 × 10^−04^	miR-24-3p	3.25 × 10^−03^	**miR-99a-5p**	5.40 × 10^−04^
**miR-423-3p**	4.79 × 10^−03^	miR-148a-3p	1.04 × 10^−03^	**miR-99a-5p**	4.74 × 10^−03^	**miR-423-3p**	6.32 × 10^−04^
miR-148a-3p	5.33 × 10^−03^	**miR-99a-5p**	1.70 × 10^−03^	miR-146a-5p	7.78 × 10^−03^	miR-9-5p	6.39 × 10^−04^
miR-150-5p	5.87 × 10^−03^	miR-150-5p	2.63 × 10^−03^	**miR-200a-3p**	8.33 × 10^−03^	miR-150-5p	8.47 × 10^−04^
miR-342-5p	6.39 × 10^−03^	miR-320d	2.90 × 10^−03^	miR-22-5p	9.21 × 10^−03^	miR-148a-3p	1.09 × 10^−03^
**miR-200a-3p**	6.55 × 10^−03^	**miR-320a**	3.26 × 10^−03^	miR-320d	9.82 × 10^−03^	miR-320d	2.86 × 10^−03^
**miR-99a-5p**	8.05 × 10^−03^	**miR-423-3p**	3.27 × 10^−03^			miR-101-3p	5.31 × 10^−03^
miR-30c-5p	8.84 × 10^−03^	miR-122-5p	7.07 × 10^−03^			miR-451a	5.62 × 10^−03^
						miR-24-3p	6.09 × 10^−03^

*CRPC, castration-resistant prostate cancer; CPM, counts per million transformation; TMM, the trimmed mean of M-value normalization. MiRNAs in bold are these differentially expression by all four analysis methods*.

### Validation of Plasma Exosomal miRNAs as Potential Biomarkers for CRPC by RT-qPCR

**Figure 2:**
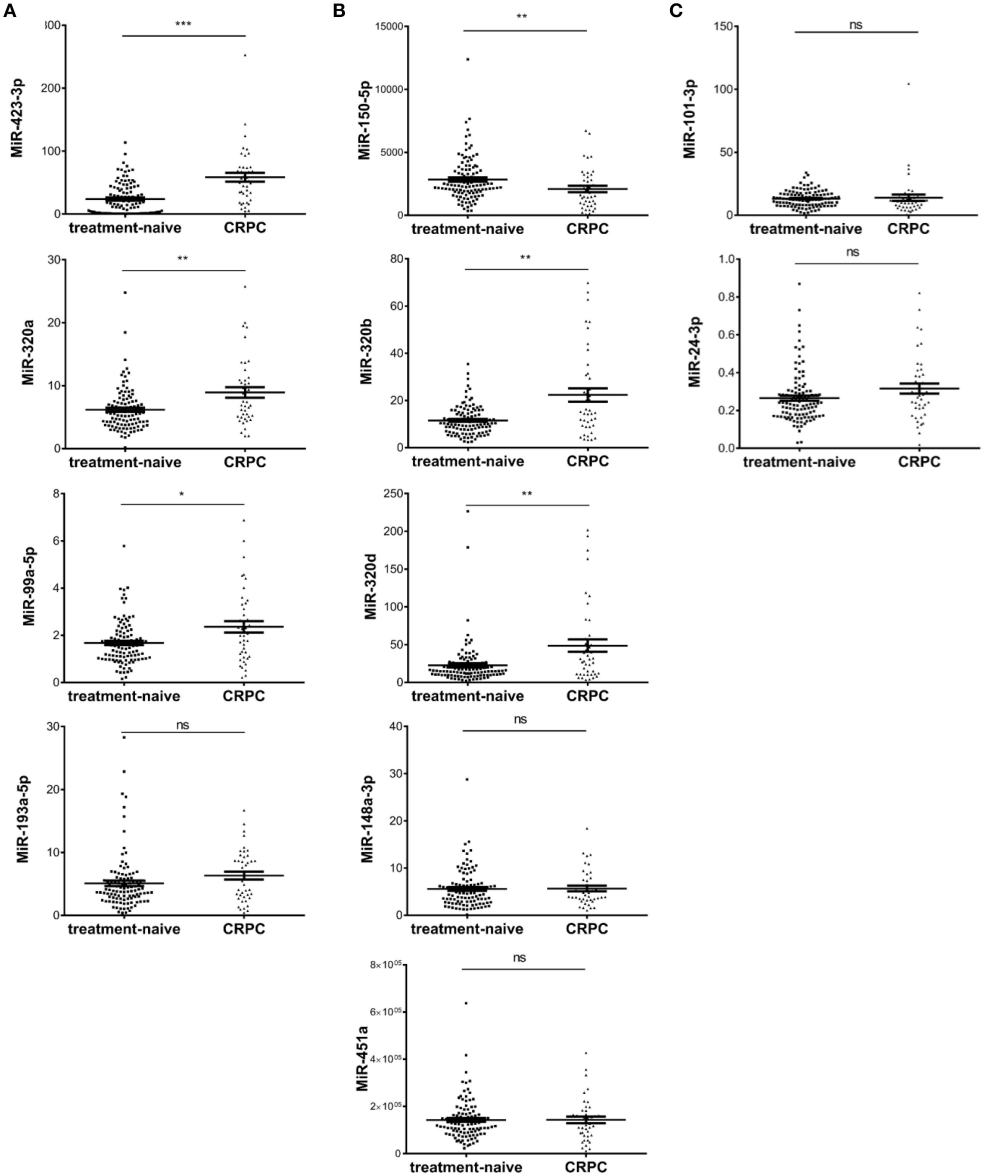
Scatter plots of plasma exosomal miRNA expression levels evaluated by RT-qPCR in validation cohort I of 108 treatment-naive prostate cancer and 42 castration resistant prostate cancer (CRPC) patients. **(A)** The five significant differentially expressed miRNAs from RNA-seq identified by four RNA-seq analysis methods; **(B)** The six significant differentially expressed miRNAs from RNA-seq identified by three RNA-seq analysis methods; **(C)** The differentially expressed miRNAs from RNA-seq identified by two RNA-seq analysis methods. The y-axis shows the relative expression level of miRNAs calculated by 2^−ΔCt^. The error bars show the mean ± standard error of mean (SEM). *p < 0.05, **p < 0.01, ***p < 0.001, ns, not significant.

To validate candidate plasma exosomal miRNAs as biomarkers for CRPC development, we performed RT-qPCR and tested all the five miRNAs with *p* < 0.01 in all four RNA-seq analysis methods, the six miRNAs with *p* < 0.01 in three methods, and three miRNAs with *p* < 0.01 in two methods in validation cohort I, consisting of 108 treatment-naive PCa and 42 CRPC patients collected in St Bartholomew's Hospital. Of the five miRNAs identified by all four sequencing data analysis methods, the expression of miR-200a-3p was too low to be detected by RT-qPCR. Out of the remaining four miRNAs, three miRNAs, miR-423-3p, miR-320a, and miR-99a-5p were significantly differentially expressed between treatment-naive PCa patients and CRPC (*p* = 7.3 × 10^−8^, 0.002 and 0.0186, respectively) (Figure 2A), while miR-193a-5p showed a trend without a statistically significant difference (*p* = 0.0547). After multiple testing correction, miR-423-3p and miR-320a remained statistically significant. Consistent with the RNA-seq data, these miRNAs were expressed at higher levels in CRPC compared to treatment-naive PCa patients.

Among the six miRNAs which showed significant difference in three of the four RNA-seq analysis methods, miR-375 could not be detected efficiently by our RT-qPCR method. Of the remaining five miRNAs, three miRNAs, miR-320d, miR-320b, and miR-150-5p were significantly differentially expressed (*p* = 0.0028, 0.0013, and 0.0058, respectively) (Figure 2B) and all remained statistically significant after multiple testing correction. miR-148a-3p and miR-451a showed no significant difference between treatment-naive PCa and CRPC (*p* = 0.95 and 0.98, respectively) (Figure 2B).

Among the three miRNAs which showed significant difference in only two of the four RNA-seq analysis methods, miR-9 had too low an expression to be detected. MiR-101-3p and miR-24-3p showed no significant difference (*p* = 0.086 and 0.075, respectively) (Figure 2C).

As treatment-naive PCa is mostly of low Gleason score (6 and 7) and more than half of the CRPC cases have a high Gleason score (8 or higher), we interrogated Gleason score associations in the treatment-naive PCa cohort, where we have sufficient number of patients for subgroup analysis. We found no significant association of Gleason score with the expression of any of the six miRNAs (Supplementary Figure 1).

**Figure 3:**
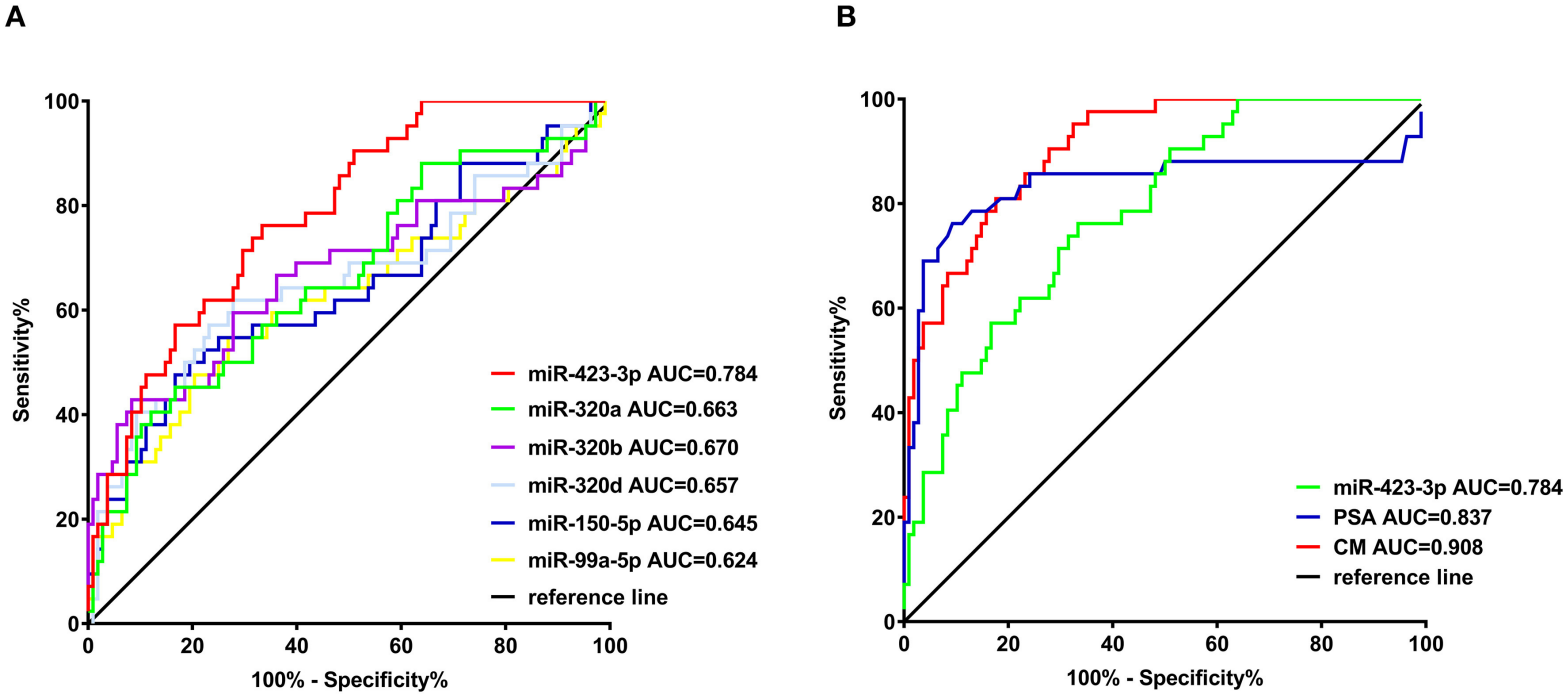
Receiver operating characteristic (ROC) analysis of the efficiencies of classifiers in discriminating castration-resistant prostate cancer (CRPC) from treatment-naive prostate cancer. **(A)** ROC analysis of the six confirmed plasma exosomal miRNAs significantly different between treatment-naive and CRPC patients in validation cohort I; **(B)** ROC analysis of miR-423-3p, serum prostate-specific antigen (PSA) and the combination model (CM) of miR-423-3p and PSA in predicting CRPC in validation cohort I.

To evaluate the predictive value of the plasma exosomal miRNAs for CRPC, we performed ROC curves analysis based on the RT-qPCR results. Comparing the treatment-naive PCa and CRPC, the AUC of miR-423-3p, miR-320a, miR-99a-5p, miR-320d, miR-320b, miR-150-5p was 0.784 (95% confidence interval (CI): 0.707–0.860, *p* < 0.0001), 0.663 (95% CI: 0.562–0.764, *p* = 0.0020), 0.624 (95% CI: 0.514–0.734, *p* = 0.019), 0.657 (95% CI: 0.549–0.766, *p* = 0.0028), 0.670 (95% CI: 0.560–0.779, *p* = 0.0013), and 0.645 (95% CI: 0.539–0.751, *p* = 0.0058), respectively (Figure 3A). When miR-423-3p, which showed the best predictive value, was combined with PSA (AUC = 0.837, 95% CI: 0.740–0.934) with a logistic binominal regression model (combination model = 0.026*PSA+0.033*miR-423-3p), the performance improved to AUC = 0.908 (95% CI: 0.861–0.955, *p* < 0.0001) with 80.95% (95% CI: 65.88–91.4%) sensitivity and 82.41% (73.9–89.06%) specificity (Figure 3B). Using a multivariate logistic regression analysis, including miR-423-3p expression and PSA level as variables, we found that both exosomal miR-423-3p expression (*p* = 7.29 × 10^−05^) and PSA level (*p* = 0.000557) were independently associated with CRPC status.

### Pathway Analysis of Differentially Expressed Exosomal miRNAs

**Figure 4:**
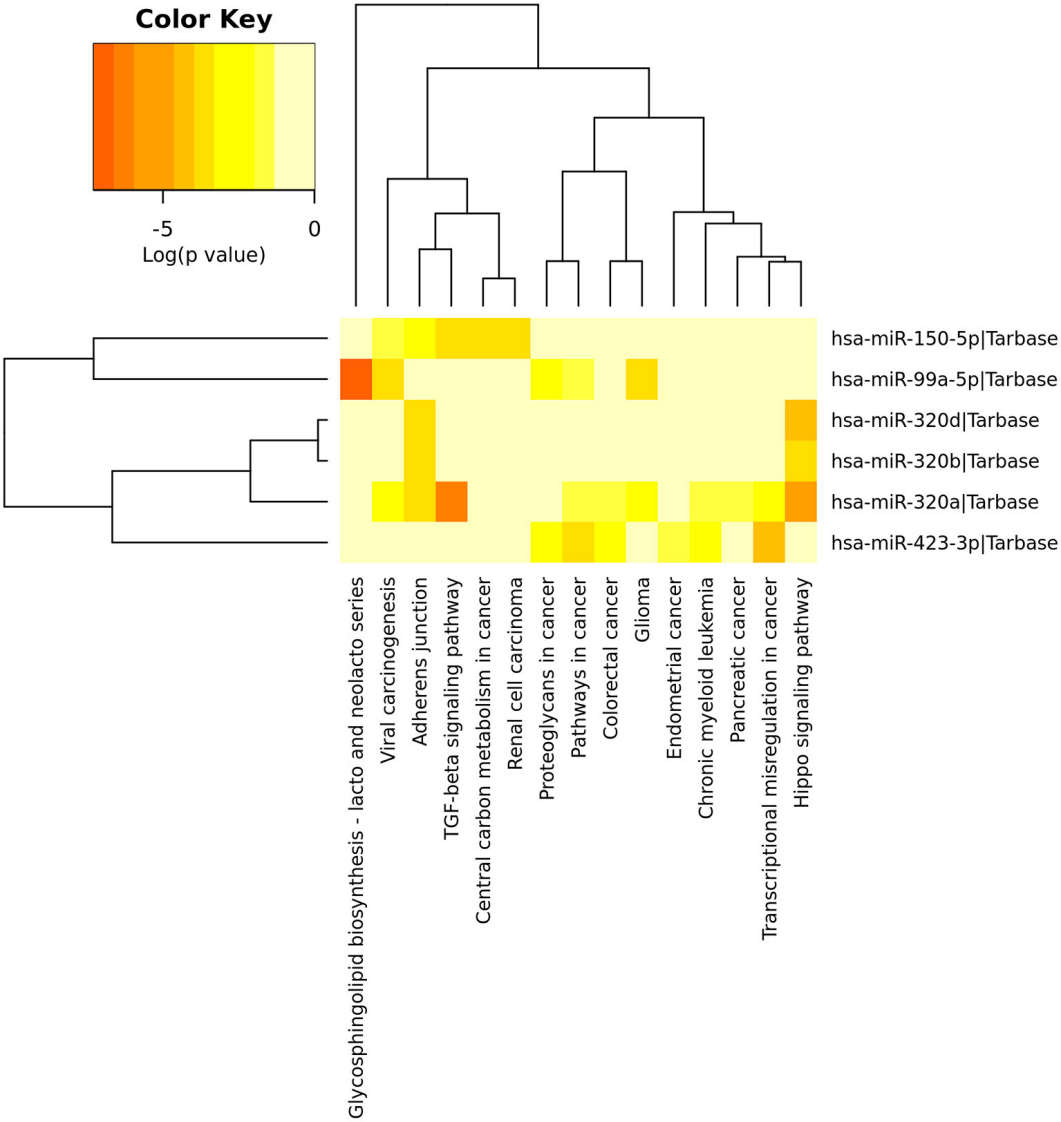
Heatmap of significant pathways predicted by DIANA-miRPath (v.3.0) for six differentially expressed plasma exosomal miRNAs between treatment-naive prostate cancer and castration-resistant prostate cancer patients. Pathways are depicted on the x-axis and miRNAs on the y-axis. The color code represents the log (*p*-value), with the most significant predicted miRNA-pathway interactions in red.

After identified plasma exosomal miRNAs associated with CRPC, we sought to explore the potential pathways in which these miRNAs are involved. We performed functional pathway analysis of targets for the six validated CRPC associated miRNAs, miR-423-3p, miR-320a, miR-99a-5p, miR-320d, miR-320b, and miR-150-5p. Targets of miRNAs were retrieved from TarBase v7.0 and pathway overrepresentation analysis was performed using KEGG pathways via DIANA-miRPath v3.0. Most of the significantly enriched pathways were cancer-associated. Hippo signaling pathway, TGF-beta signaling pathway and Adherens junction were the three most significant pathways as shown in the collected target analysis of the six differentially expressed miRNAs in Table 3. Additionally, we also explored the significantly enriched pathways (corrected *p* < 0.05) for each miRNA (Figure 4).

**Table 3:** KEGG pathway analysis of target genes of six plasma exosomal miRNAs.

**KEGG pathway**	***p*-value**	**Number of target genes**	**miRNAs**
Hippo signaling pathway	2.20 × 10^−09^	20	3
TGF-beta signaling pathway	1.71 × 10^−08^	15	2
Adherens junction	3.02 × 10^−08^	16	4
Transcriptional misregulation in cancer	8.04 × 10^−06^	32	2
Pathways in cancer	2.58 × 10^−05^	72	3
Viral carcinogenesis	5.26 × 10^−05^	43	3
Proteoglycans in cancer	6.67 × 10^−05^	25	2
Glioma	1.06 × 10^−04^	17	2
Glycosphingolipid biosynthesis—lacto and neolacto series	6.23 × 10^−04^	2	1
Colorectal cancer	1.05 × 10^−03^	16	2
Renal cell carcinoma	4.98 × 10^−03^	5	1
Central carbon metabolism in cancer	6.43 × 10^−03^	4	1
Chronic myeloid leukemia	0.012	19	2
Endometrial cancer	0.024	5	1
Pancreatic cancer	0.035	11	1

### Additional Sample Cohort Validation of Plasma Exosomal miR-423-3p as a CRPC Biomarker

**Figure 5:**
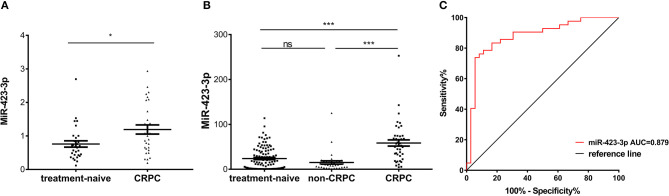
Plasma exosomal miR-423-3p expression levels evaluated by RT-qPCR in validation cohort II and treated non-CRPC. **(A)** Scatter plot of plasma exosomal miR-423-3p expression levels evaluated by RT-qPCR in validation cohort II of 30 treatment-naive prostate cancer patients and 30 castration-resistant prostate cancer (CRPC) patients; **(B)** Scatter plot of plasma exosomal miR-423-3p expression levels evaluated by RT-qPCR in treated non-CRPC patients in comparison to patients in validation cohort I; **(C)** Receiver operating characteristic (ROC) analysis of the plasma exosomal miR-423-3p in discriminating CRPC in cohort I from treated non-CRPC. In scatter plots, the y-axis show the relative expression level of miR-423-3p calculated by 2^−ΔCt^ and the error bars are showing the mean ± standard error of mean (SEM). *p<0.05, ***p < 0.001, ns, not significant.

To further validate the CRPC association of exosomal miR-423-3p, which showed the most significant difference between treatment-naive PCa and CRPC, its expression was investigated in an independent validation cohort II. This cohort consisted of 30 treatment-naive PCa and 30 CRPC patients from Mayo Clinic, where patients in the untreated PCa group were selectively enriched for metastatic disease (16/30). Plasma exosomal miRNAs were extracted by a different protocol to validation cohort I as described previously in materials and methods. Comparing the 30 treatment-naive PCa to the 30 CRPC patients, miR-423-3p was again expressed at a significantly (*p* = 0.016) higher level in plasma exosomes from CRPC than treatment-naive PCa patients (Figure 5A), which confirmed the association of plasma exosomal miR-423-3p with CRPC regardless of different cohorts and different detection methods.

### Increase of Plasma Exosomal miR-423-3p Expression Is Correlated With CRPC Development but Not in Response to ADT

To exclude the possibility that the increase of plasma exosomal miR-423-3p in CRPC as compared to treatment naive PCa is the result of ADT rather than castration resistance development, we further performed RT-qPCR analysis of plasma exosomal miR-423-3p in a group of 36 early stage treatment non-CRPC patients, who had been receiving ADT for <3 months. We found that the expression of plasma exosomal miR-423-3p was not significantly different between treatment-naive PCa and ADT-treated non-CRPC patients (*p* = 0.3353), indicating that ADT does not affect plasma exosomal miR-423-3p levels (Figure 5B). However, miR-423-3p expression was consistently and significantly (*p* < 0.0001) higher in CRPC than treated non-CRPC patients (Figure 5B). Based on the data from this cohort, plasma exosomal miR-423-3p alone had an excellent CRPC prediction value of AUC = 0.879 (95% CI: 0.7981–0.9599, *p* < 0.0001) (Figure 5C).

## Discussion

The development of CRPC is a major clinical problem in the management of advanced PCa. While a number of studies have investigated biomarkers for CRPC prognosis (Armstrong et al., 2012; Olmos et al., 2012; Ross et al., 2012; Huang et al., 2015; Pantel et al., 2019), there are limited investigations into biomarkers predicting or monitoring CRPC development (Varenhorst et al., 2016). In this study, we generated plasma exosomal miRNA profiles in treatment-naive PCa and CRPC by RNA-seq and identified plasma exosomal miRNAs associated with CRPC. We validated six differentially expressed miRNAs, miR-423-3p, miR-320a, miR-99a-5p, miR-320d, miR-320b, and miR-150-5p in a larger cohort of treatment-naive PCa and CRPC samples by RT-qPCR, with multicenter validation of miR-423-3p differential expression, which is the most significantly associated miRNA with CRPC. Furthermore, we showed that increased plasma exosomal miR-423-3p expression in CRPC is not associated with the response to ADT treatment, confirming that its expression increase is a cause or result of CRPC development. Our results show the potential of using plasma exosomal miRNAs as biomarkers to predict/monitor CRPC development, which might enable an early treatment change before CRPC is well-established.

Blood-based test has a number of advantages for clinical utility. It is non-invasive and can provide real-time information on patient status. MiRNAs in plasma exosomes serve as good candidates for blood biomarkers as they are protected from degradation, allowing for their stable and easy detection by methods such as RT-qPCR assays. In a previous study, Huang et al. generated extracellular vesicle RNA profiles from CRPC and a small number of hormone-sensitive PCa patients (Yuan et al., 2016) without exosomal miRNA analysis in association with CRPC. Using a modified plasma exosome isolation and RNA-seq method, we identified in a large patient cohort several plasma exosomal miRNAs significantly correlated to CRPC, which were validated by RT-qPCR method in a larger cohort of patients. Our data, showing that there was no significant expression difference of the six miRNAs between different Gleason grade groups, ruled out the influence of Gleason grade on the correlation of these miRNA expression with CRPC. The association of miR-423-3p with CRPC development was further supported by its differential expression in the comparison between ADT treated and CRPC cohorts and the independent validation cohort II, where the Gleason grade distribution was similar between the groups. We identified miR-423-3p as the most significantly differentially expressed miRNA between treatment-naive PCa and CRPC and the correlation of its increase with CRPC was also validated in a separate sample cohort from an independent research center. In a previous study by Watahiki et al. with a small patient cohort, higher levels of plasma miR-423-3p has been reported in patients with metastatic CRPC (n=25) compared to treatment-naive localized PCa (*n* = 25) (Watahiki et al., 2013). However, while their results are consistent with ours regarding the potential of using miR-423-3p as a circulating biomarker for metastatic CRPC prediction, we are the first to demonstrate that miR-423-3p exists in the plasma exosomes and presents at different expression levels during PCa development. This is important, as it means that plasma miR-423-3p is not just released from dying cells, but is actively secreted by cells, which can be involved in cell-cell communication to promote CRPC development. Furthermore, the result from the study by Watahiki et al. (2013) cannot rule out whether the increase of plasma miR-423-3p was the consequence of ADT treatment or CRPC development. We demonstrated that plasma exosomal miR-423-3p was associated with CRPC development instead of ADT treatment by comparing newly treated PCa patients who were at the responsive stage with those who had developed CRPC. Our study suggests that plasma exosomal miR-423-3p has strong potential to be developed in a biomarker for monitoring CRPC development and its predictive value for CRPC development should be further validated in a longitudinal study of pre-hormone therapy patients with CRPC development follow-up data.

The limited studies published to date, using patient samples to investigate genes or biomarkers associated with CRPC development, mainly compared treatment-naive PCa and CRPC samples (Nguyen et al., 2013; Watahiki et al., 2013; Goto et al., 2015). A potential issue affecting the reliability of the CRPC association is that one group of patients is untreated and the other group of patients is treated. Therefore, the genetic/molecular changes could be induced by the treatment. In this study, we have confirmed the CRPC specific association of a potential biomarker by comparing androgen depletion treated non-CRPC patients to treatment-naive PCa and CRPC patients. Therefore, we have developed a robust approach for the investigation of CRPC associated genetic changes or biomarkers using clinical samples and identified potential clinically valuable biomarkers for the early detection/prediction of CRPC occurrence.

Several normalization methods for RNA-seq data have been proposed, but no standard method has currently been established. We employed four most commonly used methods to analyze our sequencing data, CPM, Limma, TMM, and DESeq2, each with their advantages (Dillies et al., 2013; Tam et al., 2015). In our study, the miRNAs identified as differentially expressed by four and three RNA-seq analysis methods gave more reproducible results in RT-qPCR validation than those identified only by two of these methods. As there is no gold-standard method for miRNA sequencing data analysis, our results indicate that the combination of different analysis methods should be used to identify candidate miRNAs for further validation.

In addition to biomarker potential, the identified plasma exosomal miRNAs may have important functions in CRPC development. Emerging evidence shows that exosomes play a key role in cell-cell crosstalk, which may impact tumor cell growth, metastasis, angiogenesis and cancer microenvironment (Maia et al., 2018). The involvement of these six miRNAs in CRPC development has been previously reported. Consistent to our findings of miR-150-5p under-expression in CRPC patients, previous studies showed low miR-150-5p expression in CRPC tissue compared to PCa and non-PCa tissue (Okato et al., 2017) and its role as a tumor suppressor (Okato et al., 2017; Osako et al., 2017). Paradoxically, while we detected the overexpression of miR-320a, miR-320b, miR-320d, and miR-99a-5p in CRPC patient plasma exosomes, miR-320 and miR-99a-5p have been reported to suppress prostate carcinogenesis (Hsieh et al., 2013; Arai et al., 2018), and lower expression levels of miR-320a and miR-99a-5p in CRPC tissues have been reported by comparing a small number of CRPC with untreated PCa cases (Okato et al., 2016; Arai et al., 2018). The tumor suppressor role of these miRNAs does not necessarily conflict with our observations in plasma exosomes. Regarding the deregulation of these miRNAs in the metastatic CRPC tissues, these observations have to be validated in larger cohorts. The opposite results for the expression levels of miRNAs in plasma and tissue samples are not uncommon as previously found in the studies of miR-320 in glioblastoma (Roth et al., 2011; Dong et al., 2014; Manterola et al., 2014) and of miR-99a in endometrioid endometrial carcinoma (Torres et al., 2012). The pathway overrepresentation analysis identified the Hippo signaling pathway as the most significantly enriched pathway. Hippo signaling pathway has been intensively studied in cancer development which showed an important regulation role of PCa development (Salem and Hansen, 2019). Therefore, it would be interesting to further investigate if multiple miRNAs regulate Hippo signaling and cell-cell communication and play a potential role in the development of CRPC. Thus, the functional roles of these plasma exosomal miRNAs warrant further investigation.

MiR-423-3p is the most significantly dysregulated miRNA in our study. Functional studies have demonstrated the cancer promoting role of miR-423-3p in other cancer types (Guan et al., 2014; Li et al., 2015; Kong et al., 2017; Xu et al., 2018). miR-423-3p overexpression has been reported in many human cancers including lung, breast, gastric and colorectal cancers, where it acts in an oncogenic manner via enhancing cell proliferation, cell cycle progression, cell migration and invasion (Murria Estal et al., 2013; Li et al., 2015; Zhao et al., 2015; Kong et al., 2017; Sun et al., 2019; Wang et al., 2019). A study investigating brain-metastasis related miRNAs in lung adenocarcinoma found that miR-423-3p over expression directly contributed to brain metastasis by increasing cancer cell proliferation, migration and invasion (Sun et al., 2019). In Colorectal cancer, it was found that miR-423-3p was overexpressed in cancer compared to normal tissues and as a result increased cell proliferation, migration and invasion through inhibiting P21 (Li et al., 2015). However, its functional role in PCa has not yet been reported. Further studies to investigate miR-423-3p, in particular exosomal miR-423-3p, in CRPC development are warranted.

In conclusion, by analyzing the exosomal miRNA signature of multiple cohorts of treatment-naive PCa and CRPC patients with RNA-seq and RT-qPCR validation, we identified plasma exosomal miRNAs potentially associated with CRPC. Our multicenter validation as well as inclusion of androgen depletion treated non-CRPC patients confirmed the association of plasma exosomal miR-423-3p specifically with CRPC development. We demonstrated that plasma exosomal miRNAs may serve as biomarkers for non-invasive real-time monitoring of PCa status and the prediction of CRPC occurrence, which would have great potential to improve PCa treatment.

## Data Availability Statement

The datasets presented in this study can be found in online repositories. The names of the repository/repositories and accession number(s) can be found at: https://www.ncbi.nlm.nih.gov/geo/query/acc.cgi?acc=GSE136321.

## Ethics Statement

The studies involving human participants were reviewed and approved by London City & East Research Ethics Committee; Institutional Review Board of Medical College of Wisconsin. The patients/participants provided their written informed consent to participate in this study.

## Author Contributions

Y-JL and TG conceived the study. TG and YW conducted the experiments at Barts Cancer Institute. JJ conducted experiment at MCW Cancer Center. TG, JM, and Y-JL analyzed the data. XM, GSc, EB, LX, ES, SK, PR, AG, KT, JH, TO, TP, CA, GSh, WW, NF, JS, DB, MK, JL, and CD helped with sample and data collection. PR, AG, KT, JH, TO, TP, CA, GSh, WW, NF, JS, DB, LW, and Y-JL were involved in data collection and interpretation. TG, Y-JL, JM, XM, EB, LX, ES, GSh, JS, LW, and CD were involved in drafting the manuscript. Y-JL supervised the study. All authors read and approved the final manuscript.

## Conflict of Interest

The authors declare that the research was conducted in the absence of any commercial or financial relationships that could be construed as a potential conflict of interest.
