# Supplementary material for: The Identification of Plasma Exosomal miR-423-3p as a Potential Predictive Biomarker for Prostate Cancer Castration-Resistance Development by Plasma Exosomal miRNA Sequencing
Source: Front Cell Dev Biol. 2021 Jan 7;8:602493. doi: 10.3389/fcell.2020.602493 (PMC7817948; doi:10.3389/fcell.2020.602493)
Supplement: Supplementary file 2 [file Table_2.docx]

**Supplemental Table 2. Plasma exosomal miRNAs expressed in all RNA sequencing samples, treatment-naive PCa and CRPC patient samples.**

| All samples | treatment-naive PCa | CRPC |
| --- | --- | --- |
| let-7a-5p | let-7a-5p | let-7a-5p |
| let-7b-5p | let-7b-5p | let-7b-5p |
| let-7c-5p | let-7c-5p | let-7c-5p |
| let-7f-5p | let-7d-5p | let-7d-3p |
| let-7g-5p | let-7f-5p | let-7e-5p |
| let-7i-5p | let-7g-5p | let-7f-5p |
| miR-10a-5p | let-7i-5p | let-7g-5p |
| miR-10b-5p | miR-101-3p | let-7i-5p |
| miR-122-5p | miR-10a-5p | miR-10a-5p |
| miR-126-3p | miR-10b-5p | miR-10b-5p |
| miR-128-3p | miR-122-5p | miR-122-5p |
| miR-148a-3p | miR-126-3p | miR-125a-5p |
| miR-150-5p | miR-128-3p | miR-126-3p |
| miR-151a-3p | miR-148a-3p | miR-128-3p |
| miR-181a-5p | miR-150-5p | miR-143-3p |
| miR-183-5p | miR-151a-3p | miR-146a-5p |
| miR-191-5p | miR-16-5p | miR-148a-3p |
| miR-192-5p | miR-181a-5p | miR-150-5p |
| miR-21-5p | miR-183-5p | miR-151a-3p |
| miR-22-3p | miR-185-5p | miR-155-5p |
| miR-24-3p | miR-191-5p | miR-181a-5p |
| miR-25-3p | miR-192-5p | miR-183-5p |
| miR-26a-5p | miR-21-5p | miR-191-5p |
| miR-27a-3p | miR-22-3p | miR-192-5p |
| miR-30a-3p | miR-24-3p | miR-21-5p |
| miR-30d-5p | miR-25-3p | miR-22-3p |
| miR-30e-3p | miR-26a-5p | miR-24-3p |
| miR-320a | miR-27a-3p | miR-25-3p |
| miR-320b | miR-30a-3p | miR-26a-5p |
| miR-423-3p | miR-30c-5p | miR-27a-3p |
| miR-423-5p | miR-30d-5p | miR-30a-3p |
| miR-451a | miR-30e-3p | miR-30a-5p |
| miR-486-5p | miR-3168 | miR-30d-5p |
| miR-532-5p | miR-320a | miR-30e-3p |
| miR-92a-3p | miR-320b | miR-320a |
| miR-99a-5p | miR-363-3p | miR-320b |
| miR-99b-5p | miR-423-3p | miR-423-3p |
|  | miR-423-5p | miR-423-5p |
|  | miR-451a | miR-451a |
|  | miR-486-5p | miR-486-3p |
|  | miR-532-5p | miR-486-5p |
|  | miR-629-5p | miR-532-5p |
|  | miR-92a-3p | miR-92a-3p |
|  | miR-99a-5p | miR-99a-5p |
|  | miR-99b-5p | miR-99b-5p |

PCa: prostate cancer; CRPC: castration-resistant prostate cancer.
