# Supplementary material for: The Identification of Plasma Exosomal miR-423-3p as a Potential Predictive Biomarker for Prostate Cancer Castration-Resistance Development by Plasma Exosomal miRNA Sequencing
Source: Front Cell Dev Biol. 2021 Jan 7;8:602493. doi: 10.3389/fcell.2020.602493 (PMC7817948; doi:10.3389/fcell.2020.602493)
Supplement: Supplementary file 3 [file Table_3.docx]

**Supplemental Table 3. The top ten most abundant miRNAs in plasma exosomes from RNA sequencing.**

| miRNA | Read counts | | |
| --- | --- | --- | --- |
|  | Total | treatment-naive PCa | CRPC |
| miR-451a | 125536 | 65849 | 59687 |
| miR-486-5p | 63077 | 32939 | 30138 |
| let-7b-5p | 44955 | 21445 | 23510 |
| miR-126-3p | 44798 | 25910 | 18888 |
| miR-122-5p | 39275 | 6840 | 32435 |
| miR-423-5p | 35776 | 17712 | 18064 |
| miR-92a-3p | 27188 | 13134 | 14054 |
| let-7i-5p | 23056 | 9428 | 13628 |
| let-7a-5p | 22525 | 10785 | 11740 |
| miR-148a-3p | 19541 | 5696 | 13845 |

PCa: prostate cancer; CRPC: castration-resistant prostate cancer
