# Supplementary material for: The Identification of Plasma Exosomal miR-423-3p as a Potential Predictive Biomarker for Prostate Cancer Castration-Resistance Development by Plasma Exosomal miRNA Sequencing
Source: Front Cell Dev Biol. 2021 Jan 7;8:602493. doi: 10.3389/fcell.2020.602493 (PMC7817948; doi:10.3389/fcell.2020.602493)
Supplement: Supplementary file 5 [file Data_Sheet_1.docx]

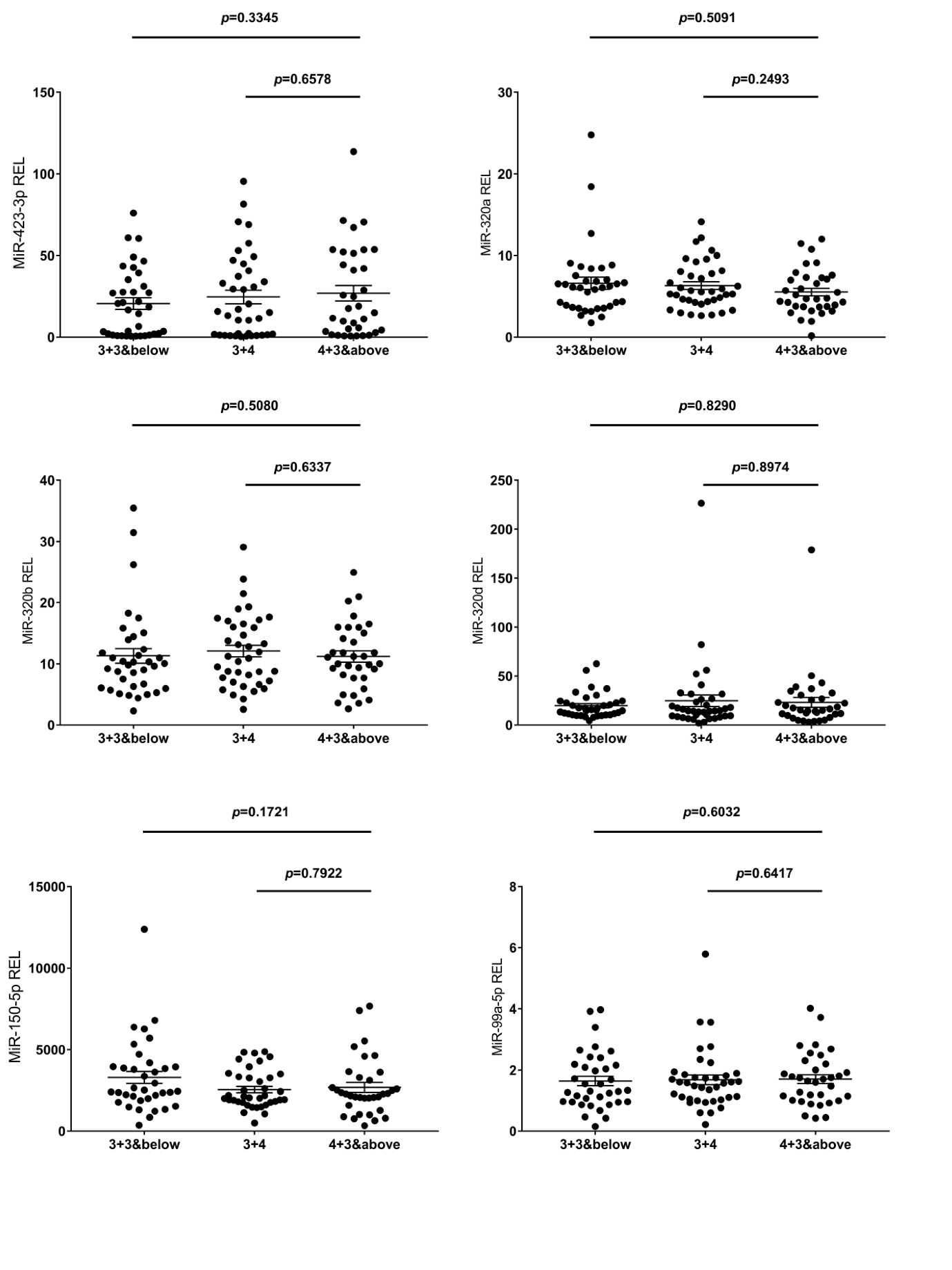


**Supplementary Figure 1. Differential expression analysis of the six miRNAs between different Gleason score groups (split into Gleason score 3+3, 3+4 and 4+3 and above groups) in the Barts treatment naïve PCa patients.** The scatter plots showed no significant difference between the Gleason groups for any miRNAs. REL: relative expression level.
